# 1,4-Bis[(2,6-dimethoxy­phen­yl)ethyn­yl]benzene

**DOI:** 10.1107/S1600536808013664

**Published:** 2008-05-14

**Authors:** Katsuhiko Ono, Kenichi Tsukamoto, Masaaki Tomura, Katsuhiro Saito

**Affiliations:** a Department of Materials Science and Engineering, Nagoya Institute of Technology, Gokiso, Showa-ku, Nagoya 466-8555, Japan; b Institute for Molecular Science, Myodaiji, Okazaki 444-8585, Japan

## Abstract

The title compound, C_26_H_22_O_4_, is a derivative of 1,4-bis­(phenyl­ethyn­yl)benzene substituted by four meth­oxy groups at the terminal benzene rings. The asymmetric unit consists of two half-molecules; one centrosymmetric molecule is planar but the other is non-planar, with dihedral angles of 67.7 (1)° between the central benzene ring and the terminal benzene rings. In the crystal structure, mol­ecules form a zigzag mol­ecular network due to π–π [the inter­planar and centroid–centroid distances between the benzene rings are 3.50 (1) and 3.57 (1) Å, respectively] and C—H⋯π inter­actions (2.75 Å). Introduction of the four meth­oxy groups results in the supra­molecular architecture.

## Related literature

The synthetic research of ethynylated aromatic compounds has attracted considerable attention because of inter­est in their mol­ecular structures (Bunz *et al.*, 1999 ▶; Kawase *et al.*, 2003 ▶), optical properties (Beeby *et al.*, 2002 ▶; Bunz, 2000 ▶) and mol­ecular electronics (Tour, 2000 ▶). 1,4-Bis(phenyl­ethyn­yl)benzene is used as a building block in applications such as liquid-crystalline materials (Dai *et al.*, 1999 ▶) and electron-conducting mol­ecular wires (Moore *et al.*, 2006 ▶). For related mol­ecular structures, including a 1,4-bis­(phenyl­ethyn­yl)benzene system, see: Watt *et al.* (2004 ▶); Li *et al.* (1998 ▶); Filatov & Petrukhina (2005 ▶).
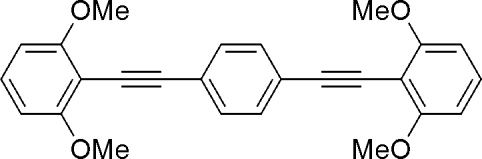


## Experimental

### 

#### Crystal data

C_26_H_22_O_4_
*M* *_r_* = 398.44
Monoclinic, 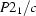

*a* = 12.391 (4) Å
*b* = 10.313 (3) Å
*c* = 16.611 (5) Å
β = 95.323 (4)°
*V* = 2113.5 (11) Å^3^
*Z* = 4
Mo *K*α radiation
μ = 0.08 mm^−1^
*T* = 173 (1) K
0.47 × 0.35 × 0.10 mm

#### Data collection

Rigaku/MSC Mercury CCD diffractometer
Absorption correction: none
16248 measured reflections
4775 independent reflections
4206 reflections with *I* > 2σ(*I*)
*R*_int_ = 0.028

#### Refinement

*R*[*F* ^2^ > 2σ(*F* ^2^)] = 0.055
*wR*(*F* ^2^) = 0.120
*S* = 1.12
4775 reflections
271 parameters
H-atom parameters constrained
Δρ_max_ = 0.20 e Å^−3^
Δρ_min_ = −0.16 e Å^−3^

### 

Data collection: *CrystalClear* (Rigaku/MSC, 2001 ▶); cell refinement: *CrystalClear*; data reduction: *CrystalClear*; program(s) used to solve structure: *SIR2004* (Burla *et al.*, 2005 ▶); program(s) used to refine structure: *SHELXL97* (Sheldrick, 2008 ▶); molecular graphics: *PLATON* (Spek, 2003 ▶) and *Mercury* (Macrae *et al.*, 2006 ▶); software used to prepare material for publication: *WinGX* (Farrugia, 1999 ▶).

## Supplementary Material

Crystal structure: contains datablocks I, global. DOI: 10.1107/S1600536808013664/xu2425sup1.cif

Structure factors: contains datablocks I. DOI: 10.1107/S1600536808013664/xu2425Isup2.hkl

Additional supplementary materials:  crystallographic information; 3D view; checkCIF report

## Figures and Tables

**Fig. 1.**
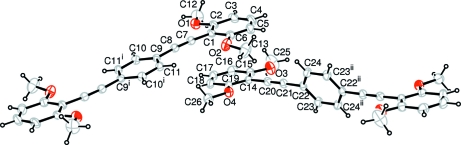
The molecular structures of (I), with atom labels and 50% probability displacement elipsoids for non-H atoms and H atoms are shown as small spheres of arbitrary radii [symmetry codes: (i) 1-x,-y,-z; (ii) 2-x,1-y,1-z].

**Fig. 2.**
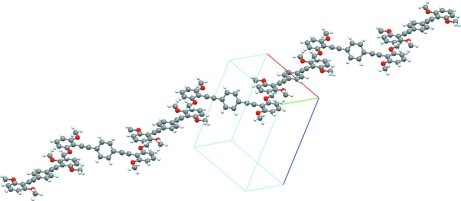
The packing diagram of (I), zigzag molecular chain.

**Fig. 3.**
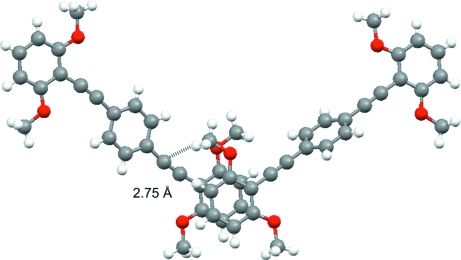
The packing diagram of (I), overlap mode.

**Fig. 4.**
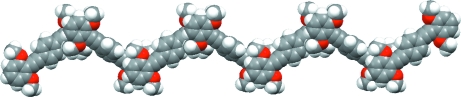
The packing diagram of (I), zigzag network.

## supplementary crystallographic information

### Comment

The synthetic research of ethynylated aromatic compounds has attracted considerable attention because of interests in their molecular structures (Bunz *et al.*, 1999; Kawase *et al.*, 2003), optical properties (Beeby *et al.*, 2002; Bunz, 2000) and molecular electronics (Tour, 2000). Among these ethynylated aromatic compounds, 1,4-bis(phenylethynyl)benzene derivatives have been extensively studied. These compounds have stiff, linear molecular structures and are used as building blocks in the applications such as liquid-crystalline materials (Dai *et al.*, 1999) and electron-conducting molecular wires (Moore *et al.*, 2006). According to the X-ray crystallographic analyses of 1,4-bis(phenylethynyl)benzene, the molecules crystallize in two crystal forms with the *P*1 and *Pbcn* space groups (Watt *et al.*, 2004; Li *et al.*, 1998). In both crystals, the molecules are linear and planar. In *P*1, the molecules are aggregated by the face-to-edge interactions based on C—H···π contacts (2.74–2.89 Å). In *Pbcn*, the molecules form π-dimers with an intermolecular distance of 3.49 Å. The π-dimers are aggregated by the face-to-face interactions based on π···π contacts (3.45 Å) and the face-to-edge interactions based on C—H···π contacts (benzene ring) (2.85–2.88 Å) and C—H···π contacts (C≡C bond) (2.79–2.87 Å). Furthermore, the X-ray crystallographic analysis carried out on 1,4-bis(*p*-tolylethynyl)benzene in *P*2_1_/*c* (Filatov & Petrukhina, 2005) again showed the molecule to be linear and planar. The molecules are stacked along the *b* axis to form a column with intermolecular distances of 3.51 and 3.56 Å. This result indicates that the introduction of two methyl groups to the terminal benzene rings provides the modification of molecular assembly. With regard to this, we investigated the molecular and crystal structure of the title compound, (I), which is a derivative substituted by four methoxy groups at the terminal benzene rings.

Single crystals of (I) were grown by recrystallization from dichloromethane. These produce a structure in *P*2_1_/*c* that shows two crystallographically independent molecules, each possessing an inversion centre (Fig. 1). One molecule is planar and strained at the C≡C bonds. The other molecule is a linear, nonplanar structure with a dihedral angle of 67.7 (1)° between the central benzene ring and the terminal benzene rings. The C≡C bond lengths are 1.200 (2) Å (C7—C8) and 1.199 (2) Å (C20—C21). These values are analogous to those of 1,4-bis(phenylethynyl)benzene (1.202–1.205 Å). The C≡C—C bond angles are 173.1 (2)° (C1—C7—C8), 174.3 (2)° (C7—C8—C9), 178.6 (2)° (C14—C20—C21) and 178.7 (2)° (C20—C21—C22). The bond angles of C1—C7—C8 and C7—C8—C9 are strained as compared to those of 1,4-bis(phenylethynyl)benzene (176.9°–179.5°). Both the molecules alternately arrange to form a zigzag molecular chain due to the π···π and C—H···π interactions (Fig. 2). The terminal benzene rings overlap each other to form a π-stacking (Fig. 3). The interplanar and centroid-centroid distances between the benzene rings are 3.50 (1) and 3.57 (1) Å, respectively. The C—H···π contact between the H26C atom and the C8 atom is observed (2.75 Å). This contact affords the strained structure at the C≡C bonds between the C7 and C8 atoms. Furthermore, the four methoxy groups lock the benzene dimer to provide the zigzag molecular network (Fig. 4). Thus, the introduction of four methoxy groups to the terminal benzene rings forms a supramolecular architecture of 1,4-bis(phenylethynyl)benzene.

In summary, we studied the molecular and crystal structure of 1,4-bis[(2,6-dimethoxyphenyl)ethynyl]benzene, which is a 1,4-bis(phenylethynyl)benzene derivative substituted by four methoxy groups at the terminal benzene rings. The methoxy groups fixed a π-stacking geometry between the terminal benzene rings resulting in the formation of the zigzag molecular network. The introduction of methoxy groups provided a supramolecular architecture of 1,4-bis(phenylethynyl)benzene.

### Experimental

The title compound (I) was prepared as follows: Bis(triphenylphosphine)palladium(II) dichloride [Pd(PPh_3_)_2_Cl_2_] (10 mg, 0.014 mmol) was added to a mixture of 1-ethynyl-2,6-dimethoxybenzene (76 mg, 0.47 mmol), 1,4-diiodobenzene (77 mg, 0.23 mmol), and copper(I) iodide (3 mg, 0.014 mmol) in dry DMF (5 ml) and dry triethylamine (5 ml) under nitrogen. The reaction mixture was stirred for 16 h at 80 °C. After removal of the solvent, dichloromethane (30 ml) and aqueous disodium ethylenediaminetetraacetate (Na_2_edta) solution (5%, 30 ml) were added. The organic layer was separated and washed with water (30 ml). The organic solution was dried over Na_2_SO_4_ and concentrated. The residue was separated by column chromatography on silica gel (CH_2_Cl_2_/hexane = 9: 1) to afford compound (I) (52 mg, 56%) as a yellow powder. Yellow crystals of (I) suitable for X-ray analysis were grown from a dichloromethane solution.

### Refinement

All the H atoms were placed in geometrically calculated positions, with C—H = 0.95 (phenyl) and 0.98 (methyl) Å, and refined using a riding model with *U*_iso_(H) = 1.2*U*_eq_(C) (phenyl) and 1.5*U*_eq_(C) (methyl).

### Crystal data

**Table:**

C_26_H_22_O_4_	*F*_000_ = 840
*M**_r_* = 398.44	*D*_x_ = 1.252 Mg m^−^^3^
Monoclinic, *P*2_1_/*c*	Melting point = 528–529 K
Hall symbol: -P 2ybc	Mo *K*α radiation λ = 0.71073 Å
*a* = 12.391 (4) Å	Cell parameters from 5564 reflections
*b* = 10.313 (3) Å	θ = 3.2–27.5º
*c* = 16.611 (5) Å	µ = 0.08 mm^−^^1^
β = 95.323 (4)º	*T* = 173 (1) K
*V* = 2113.5 (11) Å^3^	Block, yellow
*Z* = 4	0.47 × 0.35 × 0.10 mm

### Data collection

**Table:**

Rigaku/MSC Mercury CCD diffractometer	4775 independent reflections
Radiation source: rotating-anode X-ray tube	4206 reflections with *I* > 2σ(*I*)
Monochromator: Graphite Monochromator	*R*_int_ = 0.028
Detector resolution: 14.6199 pixels mm^-1^	θ_max_ = 27.5º
*T* = 173(1) K	θ_min_ = 3.2º
φ and ω scans	*h* = −16→11
Absorption correction: none	*k* = −13→11
16248 measured reflections	*l* = −21→21

### Refinement

**Table:**

Refinement on *F*^2^	Secondary atom site location: difference Fourier map
Least-squares matrix: full	Hydrogen site location: inferred from neighbouring sites
*R*[*F*^2^ > 2σ(*F*^2^)] = 0.055	H-atom parameters constrained
*wR*(*F*^2^) = 0.120	*w* = 1/[σ^2^(*F*_o_^2^) + (0.044*P*)^2^ + 0.7208*P*] where *P* = (*F*_o_^2^ + 2*F*_c_^2^)/3
*S* = 1.12	(Δ/σ)_max_ < 0.001
4775 reflections	Δρ_max_ = 0.20 e Å^−^^3^
271 parameters	Δρ_min_ = −0.16 e Å^−^^3^
Primary atom site location: structure-invariant direct methods	Extinction correction: none

### Special details

**Table:**

Experimental. IR (KBr, cm^-1^): 3002, 2836, 2209, 1582, 1514, 1474, 1429, 1300, 1258, 1113, 1034, 843, 772, 718; ^1^H NMR (CDCl_3_, δ p.p.m.): 3.92 (s, 12H), 6.56 (d, J = 8.4 Hz, 4H), 7.25 (t, J = 8.4 Hz, 2H), 7.54 (s, 4H); ^13^C NMR (CDCl_3_, δ p.p.m.): 56.1, 83.5, 97.8, 101.5, 103.5, 123.4, 130.0, 131.5, 161.5; MS (EI): m/*z* 399 (*M*^+^ + 1), 161.
Geometry. All e.s.d.'s (except the e.s.d. in the dihedral angle between two l.s. planes) are estimated using the full covariance matrix. The cell e.s.d.'s are taken into account individually in the estimation of e.s.d.'s in distances, angles and torsion angles; correlations between e.s.d.'s in cell parameters are only used when they are defined by crystal symmetry. An approximate (isotropic) treatment of cell e.s.d.'s is used for estimating e.s.d.'s involving l.s. planes.
Refinement. Refinement of *F*^2^ against ALL reflections. The weighted *R*-factor *wR* and goodness of fit *S* are based on *F*^2^, conventional *R*-factors *R* are based on *F*, with *F* set to zero for negative *F*^2^. The threshold expression of *F*^2^ > σ(*F*^2^) is used only for calculating *R*-factors(gt) *etc*. and is not relevant to the choice of reflections for refinement. *R*-factors based on *F*^2^ are statistically about twice as large as those based on *F*, and *R*- factors based on ALL data will be even larger.

### Fractional atomic coordinates and isotropic or equivalent isotropic displacement parameters (Å^2^)

**Table:**

	*x*	*y*	*z*	*U*_iso_*/*U*_eq_	
C1	0.78035 (10)	−0.11242 (15)	0.26308 (8)	0.0283 (3)	
C2	0.78339 (11)	−0.21811 (16)	0.31655 (9)	0.0316 (3)	
C3	0.86038 (12)	−0.22279 (19)	0.38320 (10)	0.0417 (4)	
H3	0.8632	−0.2949	0.4189	0.050*	
C4	0.93251 (13)	−0.1214 (2)	0.39674 (10)	0.0476 (5)	
H4	0.9849	−0.1250	0.4422	0.057*	
C5	0.93074 (12)	−0.0146 (2)	0.34601 (10)	0.0437 (4)	
H5	0.9809	0.0542	0.3567	0.052*	
C6	0.85420 (11)	−0.00986 (16)	0.27909 (9)	0.0332 (3)	
C7	0.70162 (11)	−0.10222 (14)	0.19462 (8)	0.0269 (3)	
C8	0.63967 (11)	−0.08077 (13)	0.13629 (8)	0.0270 (3)	
C9	0.56837 (10)	−0.04176 (13)	0.06729 (8)	0.0238 (3)	
C10	0.47959 (11)	−0.11676 (14)	0.03736 (8)	0.0274 (3)	
H10	0.4653	−0.1967	0.0628	0.033*	
C11	0.58769 (11)	0.07592 (14)	0.02880 (8)	0.0269 (3)	
H11	0.6476	0.1281	0.0484	0.032*	
C12	0.70206 (18)	−0.4179 (2)	0.35277 (12)	0.0591 (5)	
H12A	0.6438	−0.4770	0.3325	0.089*	
H12B	0.6878	−0.3857	0.4063	0.089*	
H12C	0.7714	−0.4643	0.3568	0.089*	
C13	0.90402 (17)	0.2050 (2)	0.24194 (13)	0.0617 (6)	
H13A	0.8873	0.2678	0.1983	0.093*	
H13B	0.9815	0.1843	0.2460	0.093*	
H13C	0.8854	0.2423	0.2931	0.093*	
C14	0.70955 (10)	0.10378 (14)	0.44408 (9)	0.0280 (3)	
C15	0.70353 (11)	0.00310 (15)	0.50009 (10)	0.0333 (3)	
C16	0.62828 (13)	−0.09645 (16)	0.48519 (11)	0.0414 (4)	
H16	0.6247	−0.1658	0.5225	0.050*	
C17	0.55901 (13)	−0.09237 (17)	0.41523 (12)	0.0451 (4)	
H17	0.5071	−0.1597	0.4054	0.054*	
C18	0.56229 (12)	0.00605 (17)	0.35890 (10)	0.0401 (4)	
H18	0.5134	0.0065	0.3114	0.048*	
C19	0.63847 (11)	0.10420 (14)	0.37318 (9)	0.0302 (3)	
C20	0.78731 (11)	0.20559 (14)	0.45872 (9)	0.0281 (3)	
C21	0.85122 (11)	0.29250 (14)	0.46985 (9)	0.0287 (3)	
C22	0.92644 (10)	0.39768 (13)	0.48466 (8)	0.0251 (3)	
C23	0.91450 (11)	0.48457 (15)	0.54763 (9)	0.0311 (3)	
H23	0.8561	0.4744	0.5803	0.037*	
C24	1.01269 (11)	0.41447 (15)	0.43720 (9)	0.0320 (3)	
H24	1.0215	0.3561	0.3941	0.038*	
C25	0.76768 (17)	−0.0831 (2)	0.62854 (14)	0.0679 (7)	
H25A	0.8233	−0.0663	0.6732	0.102*	
H25B	0.7787	−0.1696	0.6063	0.102*	
H25C	0.6958	−0.0787	0.6485	0.102*	
C26	0.58057 (15)	0.21684 (19)	0.25079 (11)	0.0494 (5)	
H26A	0.5990	0.2945	0.2209	0.074*	
H26B	0.5055	0.2231	0.2644	0.074*	
H26C	0.5886	0.1399	0.2173	0.074*	
O1	0.70668 (9)	−0.31087 (11)	0.29821 (6)	0.0383 (3)	
O2	0.84253 (9)	0.08962 (12)	0.22507 (7)	0.0428 (3)	
O3	0.77520 (9)	0.01165 (12)	0.56704 (7)	0.0462 (3)	
O4	0.65148 (8)	0.20675 (11)	0.32338 (6)	0.0374 (3)	

### Atomic displacement parameters (Å^2^)

**Table:**

	*U*^11^	*U*^22^	*U*^33^	*U*^12^	*U*^13^	*U*^23^
C1	0.0245 (6)	0.0386 (8)	0.0220 (7)	0.0056 (6)	0.0025 (5)	−0.0012 (6)
C2	0.0286 (7)	0.0411 (9)	0.0249 (7)	0.0099 (6)	0.0010 (5)	0.0001 (6)
C3	0.0373 (8)	0.0587 (11)	0.0279 (8)	0.0169 (8)	−0.0038 (6)	0.0035 (7)
C4	0.0310 (8)	0.0764 (14)	0.0334 (9)	0.0144 (8)	−0.0082 (6)	−0.0078 (9)
C5	0.0270 (7)	0.0650 (12)	0.0391 (9)	−0.0019 (7)	0.0031 (6)	−0.0161 (9)
C6	0.0294 (7)	0.0447 (9)	0.0263 (7)	0.0011 (6)	0.0077 (6)	−0.0052 (7)
C7	0.0307 (7)	0.0261 (7)	0.0240 (7)	0.0023 (5)	0.0028 (5)	0.0014 (5)
C8	0.0315 (7)	0.0251 (7)	0.0244 (7)	0.0023 (5)	0.0021 (5)	0.0016 (6)
C9	0.0260 (6)	0.0248 (7)	0.0206 (6)	0.0037 (5)	0.0023 (5)	−0.0001 (5)
C10	0.0320 (7)	0.0230 (7)	0.0272 (7)	−0.0013 (5)	0.0023 (5)	0.0050 (5)
C11	0.0267 (6)	0.0255 (7)	0.0278 (7)	−0.0030 (5)	−0.0006 (5)	0.0005 (6)
C12	0.0762 (13)	0.0491 (12)	0.0504 (12)	−0.0019 (10)	−0.0032 (10)	0.0238 (9)
C13	0.0676 (12)	0.0600 (13)	0.0596 (13)	−0.0301 (10)	0.0166 (10)	−0.0093 (10)
C14	0.0229 (6)	0.0256 (7)	0.0358 (8)	−0.0026 (5)	0.0049 (5)	−0.0056 (6)
C15	0.0274 (6)	0.0305 (8)	0.0426 (9)	−0.0034 (6)	0.0054 (6)	−0.0011 (7)
C16	0.0392 (8)	0.0315 (8)	0.0552 (11)	−0.0087 (7)	0.0129 (7)	−0.0013 (8)
C17	0.0361 (8)	0.0408 (10)	0.0597 (11)	−0.0162 (7)	0.0118 (8)	−0.0179 (9)
C18	0.0286 (7)	0.0485 (10)	0.0432 (9)	−0.0069 (7)	0.0033 (6)	−0.0195 (8)
C19	0.0254 (6)	0.0314 (8)	0.0343 (8)	0.0004 (5)	0.0056 (6)	−0.0098 (6)
C20	0.0256 (6)	0.0285 (7)	0.0300 (7)	0.0002 (5)	0.0012 (5)	−0.0003 (6)
C21	0.0274 (6)	0.0284 (7)	0.0295 (7)	−0.0016 (5)	−0.0018 (5)	0.0024 (6)
C22	0.0245 (6)	0.0252 (7)	0.0244 (7)	−0.0023 (5)	−0.0042 (5)	0.0049 (5)
C23	0.0289 (7)	0.0376 (8)	0.0271 (7)	−0.0072 (6)	0.0044 (5)	−0.0015 (6)
C24	0.0325 (7)	0.0346 (8)	0.0289 (7)	−0.0055 (6)	0.0031 (6)	−0.0072 (6)
C25	0.0531 (11)	0.0773 (16)	0.0714 (14)	−0.0161 (10)	−0.0051 (10)	0.0420 (13)
C26	0.0521 (10)	0.0511 (11)	0.0417 (10)	0.0142 (8)	−0.0138 (8)	−0.0071 (8)
O1	0.0445 (6)	0.0364 (6)	0.0326 (6)	0.0021 (5)	−0.0033 (5)	0.0101 (5)
O2	0.0515 (7)	0.0427 (7)	0.0349 (6)	−0.0140 (5)	0.0081 (5)	−0.0049 (5)
O3	0.0429 (6)	0.0470 (7)	0.0468 (7)	−0.0138 (5)	−0.0057 (5)	0.0164 (6)
O4	0.0366 (5)	0.0418 (7)	0.0326 (6)	0.0005 (5)	−0.0035 (4)	−0.0025 (5)

### Geometric parameters (Å, °)

**Table:**

C1—C2	1.404 (2)	C14—C15	1.401 (2)
C1—C6	1.408 (2)	C14—C19	1.404 (2)
C1—C7	1.4318 (19)	C14—C20	1.4306 (19)
C2—O1	1.3630 (19)	C15—O3	1.3601 (19)
C2—C3	1.394 (2)	C15—C16	1.394 (2)
C3—C4	1.380 (3)	C16—C17	1.380 (3)
C3—H3	0.9500	C16—H16	0.9500
C4—C5	1.386 (3)	C17—C18	1.384 (3)
C4—H4	0.9500	C17—H17	0.9500
C5—C6	1.394 (2)	C18—C19	1.389 (2)
C5—H5	0.9500	C18—H18	0.9500
C6—O2	1.362 (2)	C19—O4	1.3614 (19)
C7—C8	1.1996 (19)	C20—C21	1.199 (2)
C8—C9	1.4382 (18)	C21—C22	1.4364 (19)
C9—C10	1.3979 (19)	C22—C23	1.396 (2)
C9—C11	1.4026 (19)	C22—C24	1.3964 (19)
C10—C11^i^	1.3821 (19)	C23—C24^ii^	1.385 (2)
C10—H10	0.9500	C23—H23	0.9500
C11—C10^i^	1.3821 (19)	C24—C23^ii^	1.385 (2)
C11—H11	0.9500	C24—H24	0.9500
C12—O1	1.433 (2)	C25—O3	1.423 (2)
C12—H12A	0.9800	C25—H25A	0.9800
C12—H12B	0.9800	C25—H25B	0.9800
C12—H12C	0.9800	C25—H25C	0.9800
C13—O2	1.427 (2)	C26—O4	1.4284 (19)
C13—H13A	0.9800	C26—H26A	0.9800
C13—H13B	0.9800	C26—H26B	0.9800
C13—H13C	0.9800	C26—H26C	0.9800
			
C2—C1—C6	119.00 (13)	C19—C14—C20	120.04 (13)
C2—C1—C7	122.39 (13)	O3—C15—C16	124.64 (15)
C6—C1—C7	118.55 (13)	O3—C15—C14	115.09 (13)
O1—C2—C3	124.40 (15)	C16—C15—C14	120.27 (15)
O1—C2—C1	115.23 (12)	C17—C16—C15	118.74 (16)
C3—C2—C1	120.37 (15)	C17—C16—H16	120.6
C4—C3—C2	119.23 (16)	C15—C16—H16	120.6
C4—C3—H3	120.4	C16—C17—C18	122.47 (15)
C2—C3—H3	120.4	C16—C17—H17	118.8
C3—C4—C5	121.97 (15)	C18—C17—H17	118.8
C3—C4—H4	119.0	C17—C18—C19	118.76 (15)
C5—C4—H4	119.0	C17—C18—H18	120.6
C4—C5—C6	118.97 (16)	C19—C18—H18	120.6
C4—C5—H5	120.5	O4—C19—C18	125.39 (14)
C6—C5—H5	120.5	O4—C19—C14	114.32 (12)
O2—C6—C5	125.10 (15)	C18—C19—C14	120.29 (15)
O2—C6—C1	114.47 (13)	C21—C20—C14	178.63 (16)
C5—C6—C1	120.43 (15)	C20—C21—C22	178.69 (16)
C8—C7—C1	173.14 (15)	C23—C22—C24	118.91 (12)
C7—C8—C9	174.33 (15)	C23—C22—C21	120.06 (12)
C10—C9—C11	118.60 (12)	C24—C22—C21	121.03 (13)
C10—C9—C8	122.25 (13)	C24^ii^—C23—C22	120.42 (13)
C11—C9—C8	119.15 (12)	C24^ii^—C23—H23	119.8
C11^i^—C10—C9	120.72 (13)	C22—C23—H23	119.8
C11^i^—C10—H10	119.6	C23^ii^—C24—C22	120.68 (13)
C9—C10—H10	119.6	C23^ii^—C24—H24	119.7
C10^i^—C11—C9	120.68 (13)	C22—C24—H24	119.7
C10^i^—C11—H11	119.7	O3—C25—H25A	109.5
C9—C11—H11	119.7	O3—C25—H25B	109.5
O1—C12—H12A	109.5	H25A—C25—H25B	109.5
O1—C12—H12B	109.5	O3—C25—H25C	109.5
H12A—C12—H12B	109.5	H25A—C25—H25C	109.5
O1—C12—H12C	109.5	H25B—C25—H25C	109.5
H12A—C12—H12C	109.5	O4—C26—H26A	109.5
H12B—C12—H12C	109.5	O4—C26—H26B	109.5
O2—C13—H13A	109.5	H26A—C26—H26B	109.5
O2—C13—H13B	109.5	O4—C26—H26C	109.5
H13A—C13—H13B	109.5	H26A—C26—H26C	109.5
O2—C13—H13C	109.5	H26B—C26—H26C	109.5
H13A—C13—H13C	109.5	C2—O1—C12	117.87 (13)
H13B—C13—H13C	109.5	C6—O2—C13	118.48 (14)
C15—C14—C19	119.45 (13)	C15—O3—C25	117.46 (14)
C15—C14—C20	120.51 (13)	C19—O4—C26	118.12 (13)
			
C6—C1—C2—O1	177.73 (12)	O3—C15—C16—C17	179.04 (15)
C7—C1—C2—O1	0.57 (19)	C14—C15—C16—C17	−1.2 (2)
C6—C1—C2—C3	−1.7 (2)	C15—C16—C17—C18	0.8 (2)
C7—C1—C2—C3	−178.89 (13)	C16—C17—C18—C19	0.3 (2)
O1—C2—C3—C4	−178.42 (14)	C17—C18—C19—O4	179.83 (14)
C1—C2—C3—C4	1.0 (2)	C17—C18—C19—C14	−0.8 (2)
C2—C3—C4—C5	0.1 (2)	C15—C14—C19—O4	179.83 (12)
C3—C4—C5—C6	−0.4 (2)	C20—C14—C19—O4	−0.16 (18)
C4—C5—C6—O2	179.10 (14)	C15—C14—C19—C18	0.4 (2)
C4—C5—C6—C1	−0.4 (2)	C20—C14—C19—C18	−179.56 (13)
C2—C1—C6—O2	−178.13 (12)	C15—C14—C20—C21	−160 (7)
C7—C1—C6—O2	−0.85 (18)	C19—C14—C20—C21	20 (7)
C2—C1—C6—C5	1.4 (2)	C14—C20—C21—C22	91 (11)
C7—C1—C6—C5	178.71 (13)	C20—C21—C22—C23	2(7)
C2—C1—C7—C8	171.6 (11)	C20—C21—C22—C24	−179 (100)
C6—C1—C7—C8	−5.6 (12)	C24—C22—C23—C24^ii^	−0.2 (2)
C1—C7—C8—C9	−13 (2)	C21—C22—C23—C24^ii^	179.38 (13)
C7—C8—C9—C10	−158.3 (14)	C23—C22—C24—C23^ii^	0.2 (2)
C7—C8—C9—C11	21.2 (15)	C21—C22—C24—C23^ii^	−179.37 (14)
C11—C9—C10—C11^i^	0.1 (2)	C3—C2—O1—C12	2.6 (2)
C8—C9—C10—C11^i^	179.59 (13)	C1—C2—O1—C12	−176.88 (14)
C10—C9—C11—C10^i^	−0.1 (2)	C5—C6—O2—C13	−7.5 (2)
C8—C9—C11—C10^i^	−179.60 (12)	C1—C6—O2—C13	171.99 (14)
C19—C14—C15—O3	−179.60 (13)	C16—C15—O3—C25	−4.4 (2)
C20—C14—C15—O3	0.4 (2)	C14—C15—O3—C25	175.75 (16)
C19—C14—C15—C16	0.6 (2)	C18—C19—O4—C26	1.0 (2)
C20—C14—C15—C16	−179.42 (14)	C14—C19—O4—C26	−178.37 (13)

Symmetry codes: (i) −*x*+1, −*y*, −*z*; (ii) −*x*+2, −*y*+1, −*z*+1.
